# Supplementary material for: Context-dependent selectivity to natural images in the retina
Source: Nat Commun. 2022 Sep 22;13:5556. doi: 10.1038/s41467-022-33242-8 (PMC9499945; doi:10.1038/s41467-022-33242-8)
Supplement: Supplementary file 1 — Supplementary Information [file 41467_2022_33242_MOESM1_ESM.pdf]

Supplementary Information for:

Title

Context-dependent selectivity to natural images in the retina

Authors

Goldin, Matías A.[1†*] ; Lefebvre, Baptiste[1,2†] ; Virgili, Samuele[1†] ; Pham Van Cang, Mathieu Kim[1,3]; Ecker, Alexander[4] ; Mora, Thierry[2] ; Ferrari, Ulisse[1] ; Marre, Olivier[1*]

Affiliations

1 Institut de la Vision, Sorbonne Université, INSERM, CNRS, Paris
2 Laboratoire de physique de l'Ecole normale supérieure, CNRS, PSL University, Sorbonne University, and University of Paris, Paris, France
3 Institut de l'Audition, Institut Pasteur, INSERM, Paris, France
4 Institute of Computer Science and Campus Institute Data Science, University of Göttingen, Germany

Footnotes

[†]: equal contributions
*: corresponding authors: matias.goldin@inserm.fr olivier.marre@inserm.fr

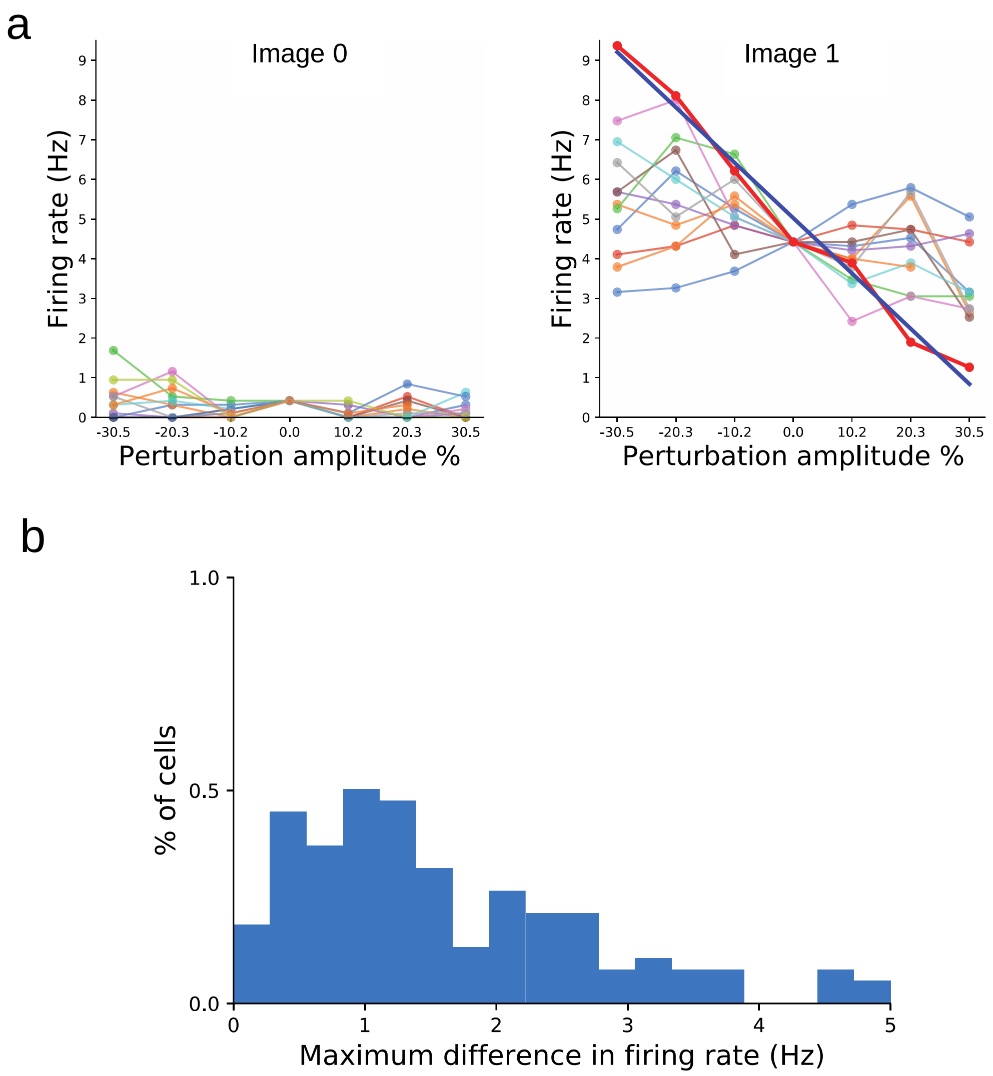

Supplementary Figure 1. Calibration for the perturbative approach amplitude.

(A) Four natural images were selected as reference, and 12 perturbation patterns were added to them using 6 different amplitudes (an amplitude of 100% would correspond to adding an amplitude equal to the mean luminance of the image). Each perturbed image was displayed to a mouse retina 25 times. All presentations were interleaved. The two panels show the 12 response curves for two images and each perturbation for an example ganglion cell, as a function of the perturbation amplitude. The response was estimated by counting spikes in a time window from 30 ms to 350 ms after image presentation. Here, the ganglion cell responds mostly to Image 1. For each curve, we fitted a linear regression and calculated the slope. We picked the perturbation having the highest slope, across all images, for each cell. We used this maximal slope to estimate, for a given perturbation amplitude, the difference between the firing rate evoked by the "maximal" perturbation, and the response to the image alone.

(B) Distribution of the maximal change in firing rate as estimated above, across many ganglion cells (n=140), for a perturbation amplitude of 25%. This amplitude gave us an average change of 1.7 Hz, corresponding to an average change in the spike count of 0.5 in the time window used to estimate the response. The selection criteria for the value of 32 was that we obtained at least 0.5 mean spike variation with respect to the non-perturbed image for each perturbed image presentation. A value of 12.5% was selected for salamander in the same manner.

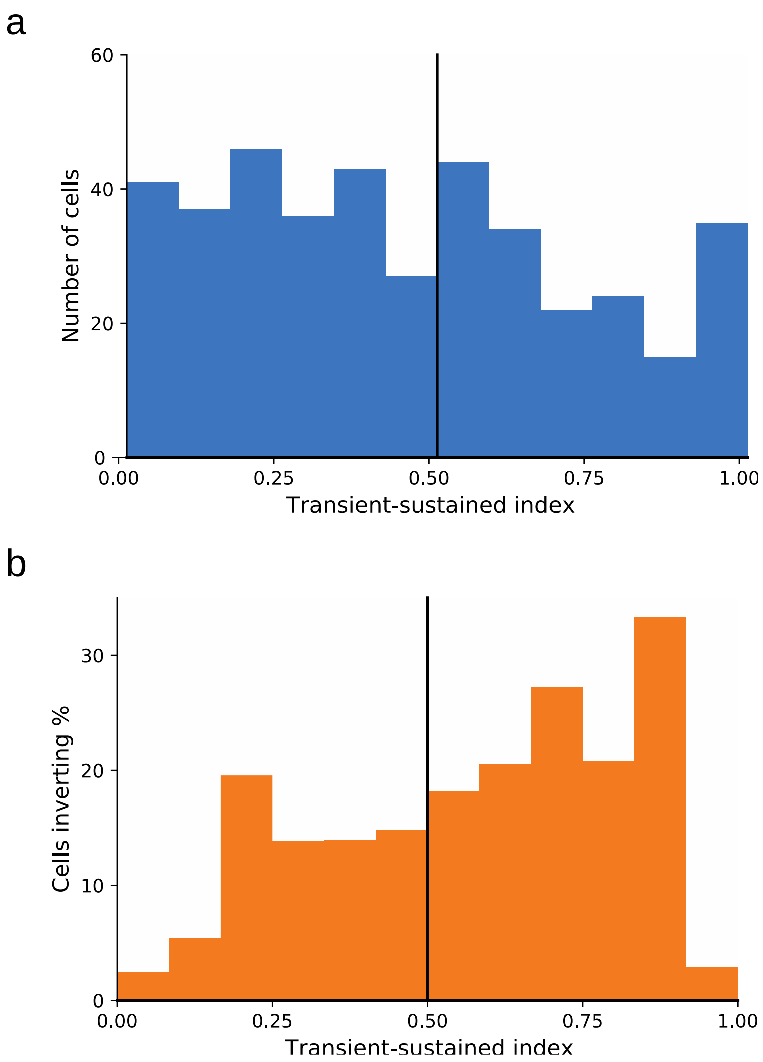

Supplementary Figure 2. Polarity inverting ganglion cells are more transient.

(A) Distribution of the Transient-Sustained index computed for all mouse retinal ganglion cells. We estimated the index from ganglion responses to a full-field flash. The early response E was estimated as the number of spikes emitted from 0 to 0.8 s after the beginning of the stimulus. The late part of the response L was estimated as the average spike count from 0.8 to 1.6 s after the onset of the stimulus. The index is defined as: (E-L)/(E+L). If the index is 1 it means that all the spikes occurred in the early window, thus indicating a transient response. On the contrary, if the index is near 0, it means that there are as many spikes in the late window as in the early one, indicating a sustained response.

(B) For each of the bins in A, we calculated the percentage of cells that showed polarity inversion during our perturbative approach.

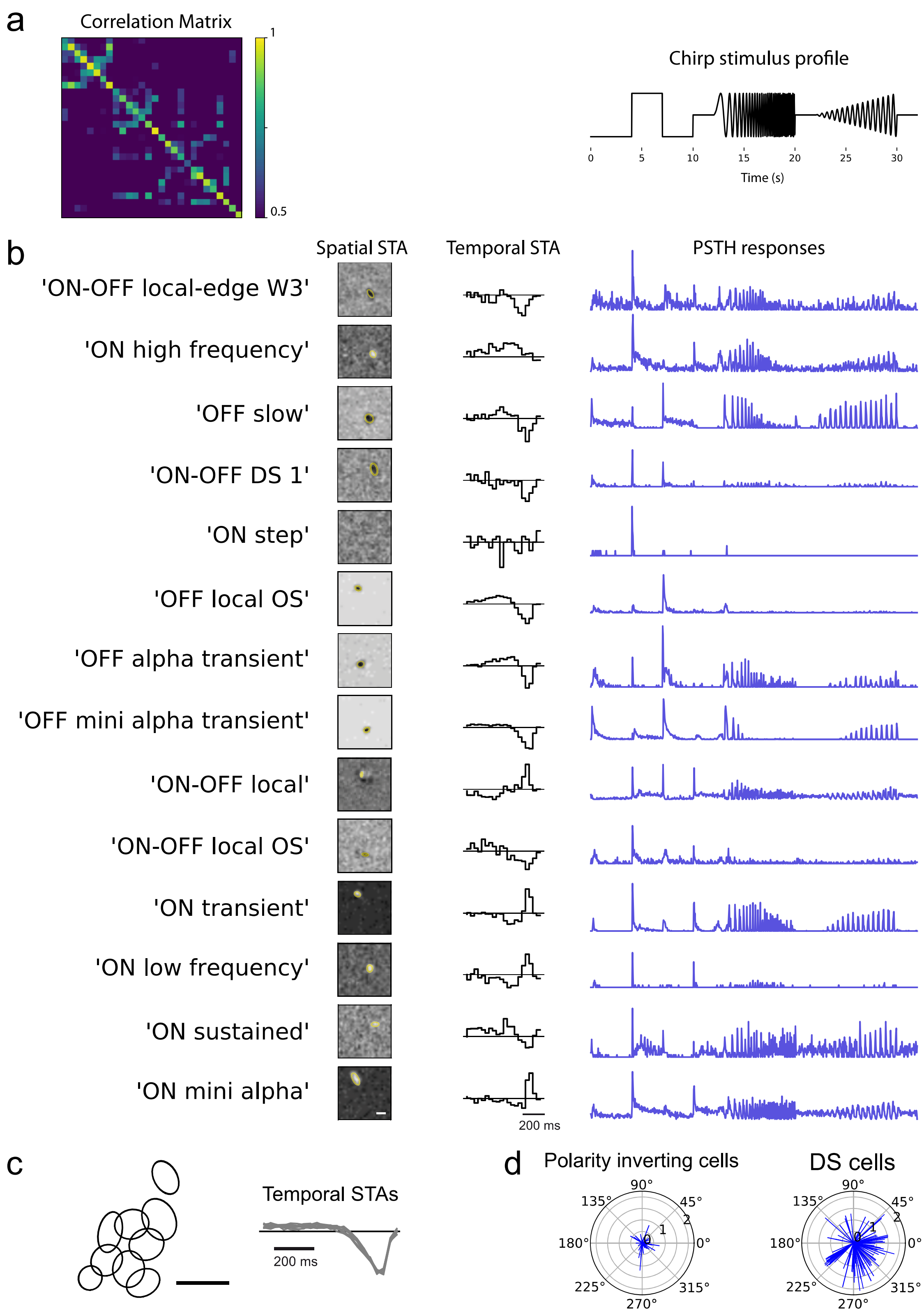

Supplementary Figure 3. Ganglion cell types.

(A) Correlation matrix showing homogeneity of the clustered cells. Each cell within a group was correlated with all other 29 cell type groups average PSTH we found (see Supplementary Table 1). The matrix shows the average of this values.

(B) Example cells that showed polarity inversion. All cells contain a detectable ON and OFF component in their responses, which is seen at the onset of the ON light step and OFF light step. Cells with pure ON or OFF responses do not show polarity inversion. Grayscale for spatial STAs denote polarity, dark are OFF and bright are ON responses. Scale bar: 200μm.

(C) Example data set from a cell type group that was used to assess the homogeneity of the clustered cell types. This group, the ON mini alpha transient, shows a clear mosaic, overlapping Temporal STAs (gray curves), and absence of directional tuning (see Methods). On the contrary groups with undetectable OFF responses, (e.g., the ON alpha type, with pure ON responses) did not have any polarity inverting cell. The same applied to groups with undetectable ON responses (pure OFF). Scale bar: 200μm.

(D) The polar plots show with blue lines the vector sum of the normalized firing rate responses of cells to drifting gratings in 8 directions (see Methods). Left: All ganglion cells where we found polarity inversion. Right: All ganglion cells that were detected to be directionally selective (see Methods).

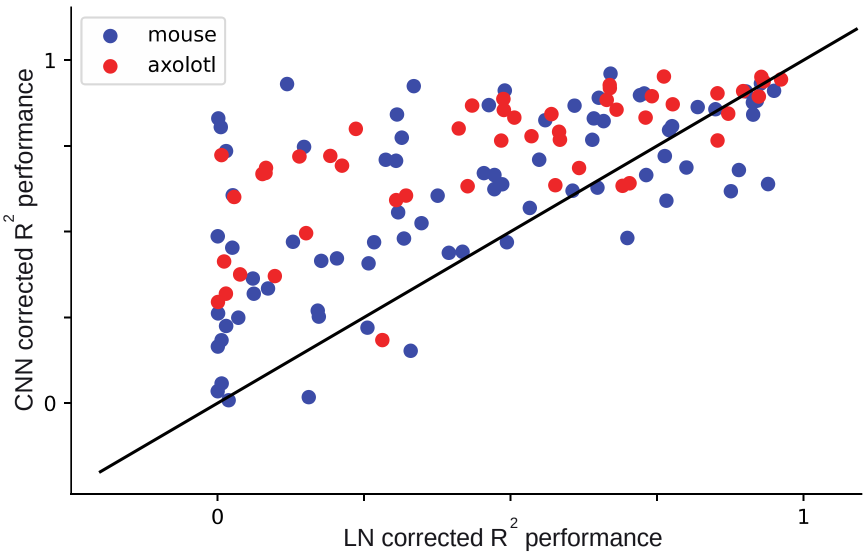

Supplementary Figure 4. Scatter plot of model performance for all cells, in reference to Fig.2
The model performance of the CNN was higher than the LN performance in most of the cells that we model, both in mouse (blue) and axolotl (red).

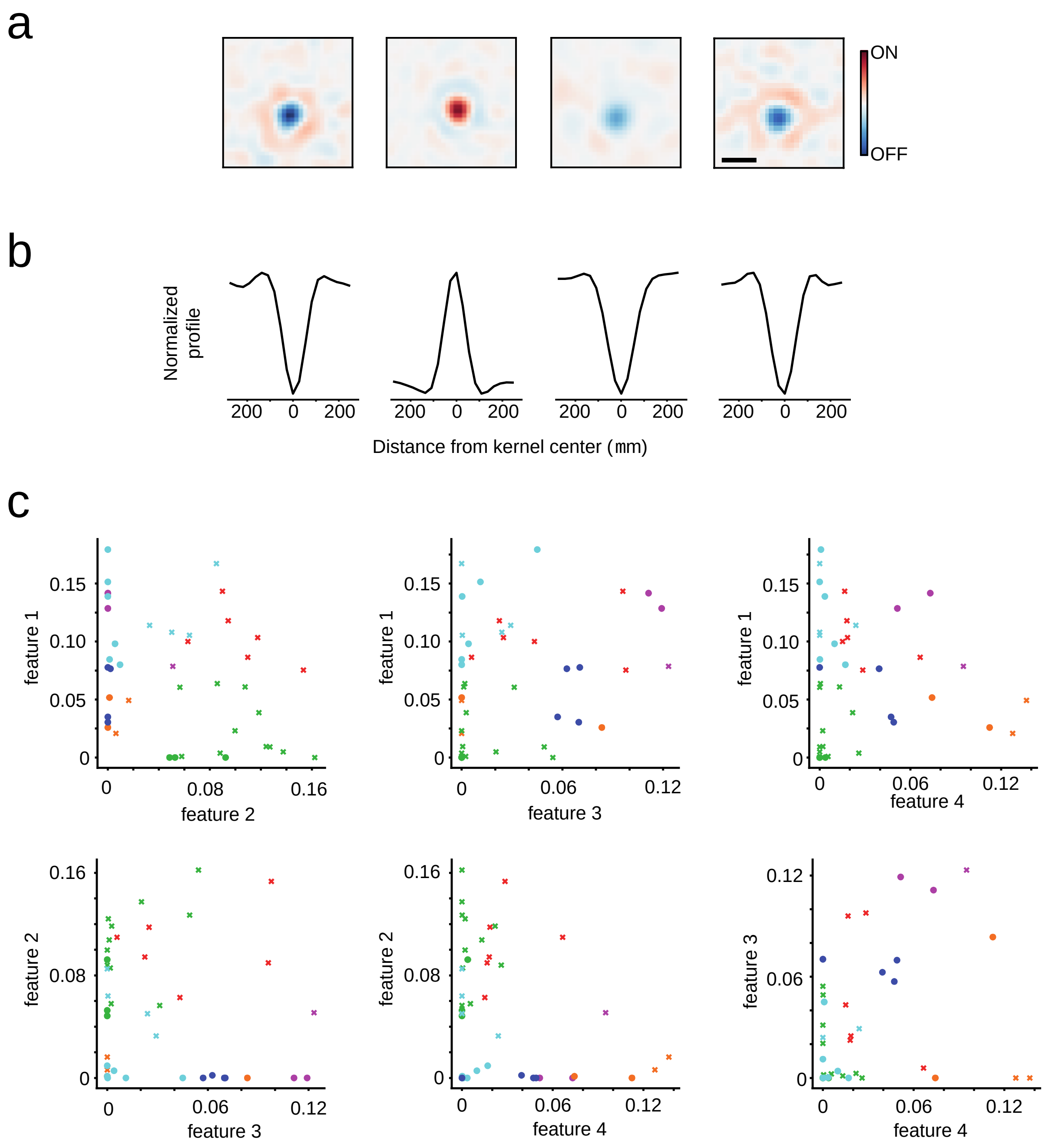

Supplementary Figure 5. Convolutional kernels and scatter plots of model features, in reference to Fig. 3.

(A) The four kernels of the model displayed in Fig. 3A. Scale bar 200 μm.

(B) Average profile calculated from the center of each kernel across every angle. The kernels display a clear opposing polarity surround.

(C) Scatter plots of model features of 41 modeled cells for one example retina. The feature weights here correspond to the kernels in A. Cells labeled with crosses are polarity inverting. For visualization purposes, the 4 features were scatter-plotted in pairs. The different colors represent different putative clusters that were obtained with standard clustering techniques. We could not obtain any clear cluster separation.

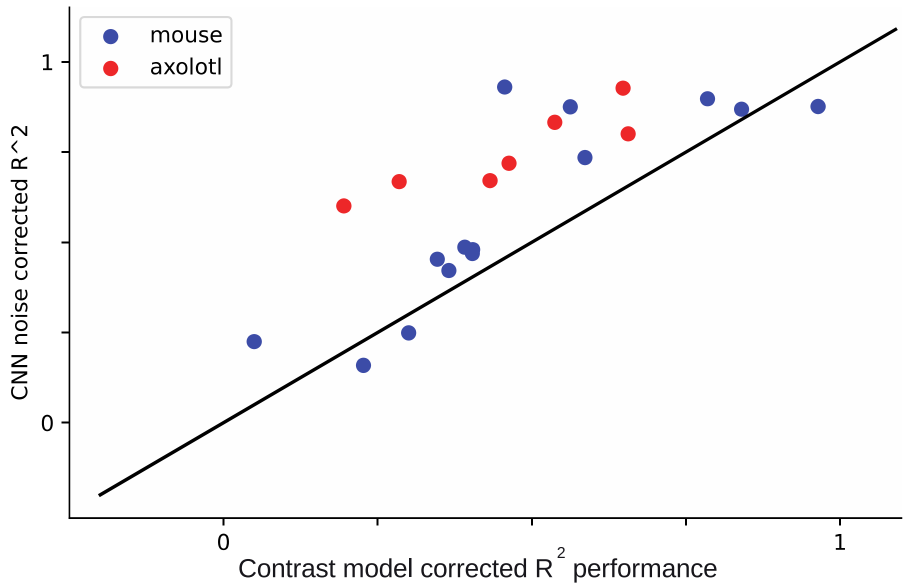

Supplementary Figure 6. Scatter plot of CNN vs contrast model performance, in reference to Fig. 4.

The model performance of the CNN was higher than the contrast model performance in most of the cells that we model, both in mouse (blue) and axolotl (red), even though the contrast model captures the qualitative behavior of these cells (Fig. 4).

a

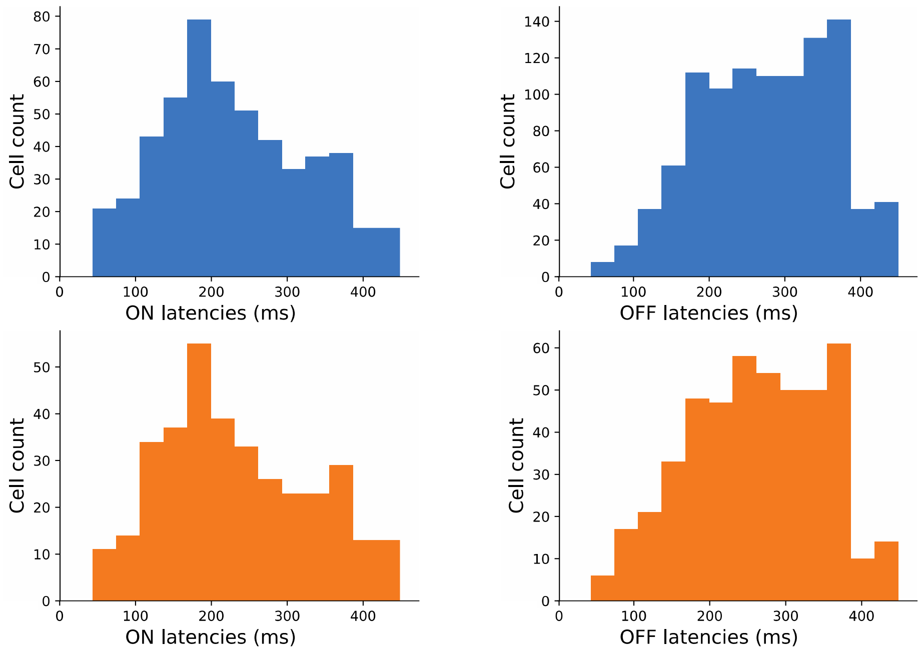

b

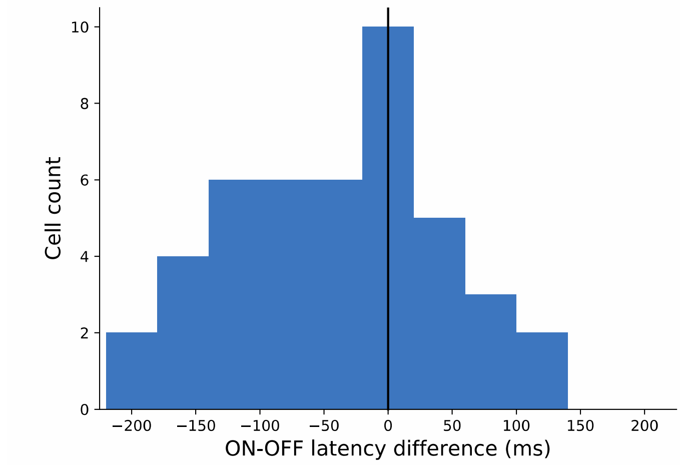

Supplementary Figure 7. Latency difference between ON an OFF LSTAs for the polarity inverting cells.

(A) We calculated the ON latency (left column) and OFF latency (right column) for all mouse retinal ganglion cells (top row, blue) for the perturbed images where an LSTA was detected. To smooth the average responses, each spike after a stimulus was replaced by a Gaussian centered at the time of the spike with a standard deviation of 20 ms, and then all the curves were averaged to obtain the average response to the perturbations. Then, we looked for the peak of the resulting curve in a window starting from 30 ms to 450 ms after the perturbed image presentation. We did the same for the polarity inverting retinal ganglion cells (bottom row, orange), with no noticeable difference with the whole population.

(B) Difference in latency between the responses associated to ON vs OFF LSTAs for the retinal ganglion cells showing polarity inversion. The peak of the distribution is at 0, but the long tails of the distribution of latencies go from -220ms to +140 ms.

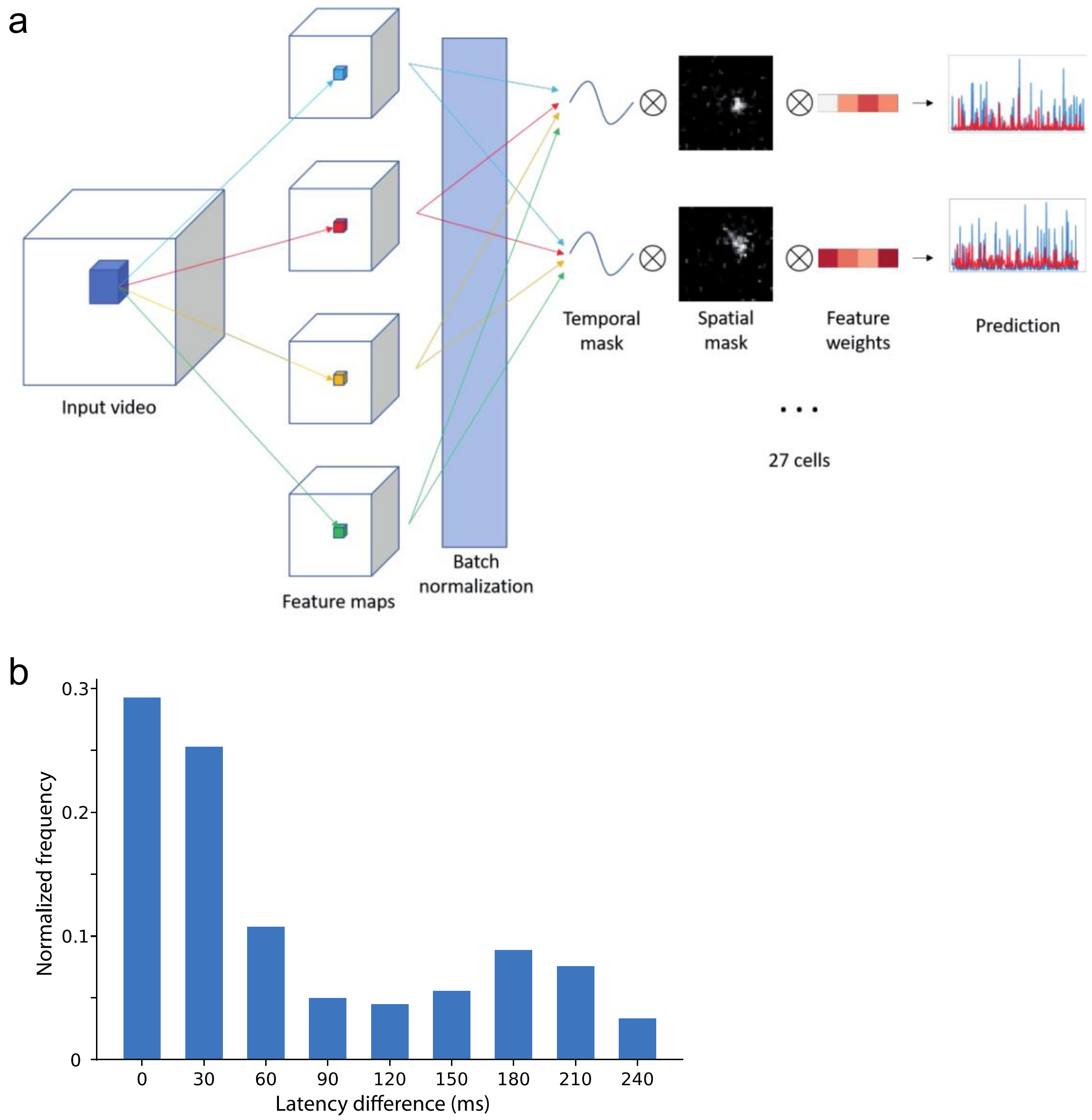

Supplementary Figure 8. Extended CNN model to account for time.
(A) The modified CNN model: For the first layer it has several kernels as our original CNN, but we switch from 2D to 3D kernels (two spatial and one time dimension). For the second layer, we add a temporal mask to the spatial mask in order to integrate inputs over time besides space. We transform each input natural image into a movie composed of 10 successive frames, all identical to the starting natural image. The output for each cell is the image response spike count, divided in 30 ms time bins. Therefore, a single spike count number from our original CNN, which integrated 300 ms, becomes a vector with 10 entries in the new framework. We trained the model to predict spike counts at a given time bin using the input at previous time bins. The response latency was detected as the bin presenting the response onset, defined as the bin in which the first PSTH maximum was observed, after smoothing.
(B) When tested on the repeated images test set, this model gave heterogeneous prediction performances. In 40% of the cases, the latency difference with the real data was predicted with an error above 60 ms. The performance of this model strongly depended on the cell, and for some cells, it made important errors.

Group Type	# Cells detected	# Cells inverting	% Cells inverting
**OFF local OS**	21	6	**29%**
OFF DS	0	-	**-**
OFF step	15	0	0%
**OFF slow**	33	1	**3%**
OFF alpha sustained	19	0	0%
ON-OFF JAM-B	21	0	0%
OFF sustained	9	0	0%
**OFF alpha transient**	13	3	**23%**
**OFF mini alpha transient**	31	6	**19%**
**ON-OFF local-edge W3**	21	2	**10%**
**ON-OFF local**	18	1	**6%**
**ON-OFF DS 1**	14	1	**7%**
ON-OFF DS 2	21	0	0%
**ON-OFF local OS**	33	4	**12%**
**ON step**	28	3	**11%**
ON DS transient	7	0	0%
ON local transient OS	34	0	0%
**ON transient**	19	3	**16%**
ON transient large	0	-	-
**ON high frequency**	13	1	**8%**
**ON low frequency**	26	1	**4%**
**ON sustained**	27	2	**7%**
**ON mini alpha**	27	2	**7%**
ON alpha	21	0	0%
ON DS sustained 1	2	0	0%
ON DS sustained 2	0	-	-
ON slow	37	0	0%
ON contrast suppression	8	0	0%
ON DS sustained 3	3	0	0%
ON local sustained OS	16	0	0%
OFF suppression 1	13	0	0%
OFF suppression 2	6	0	0%
Total number of cells	556	36	6%

Supplementary Table 1. Polarity inverting cells can belong to many different cell types. Cell type classification from the chirp stimulus and the checkerboard stimulus (See Methods). The polarity inverting cells do not belong to a particular cell type, but may belong to many. We found polarity inverting cells in 14 out of 29 types of cells detected. The lower percentage of total inverting cells reported here is because we take into account all the cells that respond to the typing stimulus, including the ones that do not present LSTAs to our perturbative approach.
