# Supplementary material for: Context-dependent selectivity to natural images in the retina
Source: Nat Commun. 2022 Sep 22;13:5556. doi: 10.1038/s41467-022-33242-8 (PMC9499945; doi:10.1038/s41467-022-33242-8)
Supplement: Supplementary file 3 — Reporting Summary [file 41467_2022_33242_MOESM3_ESM.pdf]

Corresponding author(s): Olivier Marre & Matias Goldin

Last updated by author(s): Jul 21, 2022

Reporting Summary

Nature Portfolio wishes to improve the reproducibility of the work that we publish. This form provides structure for consistency and transparency in reporting. For further information on Nature Portfolio policies, see our Editorial Policies and the Editorial Policy Checklist.

Statistics

For all statistical analyses, confirm that the following items are present in the figure legend, table legend, main text, or Methods section.

n/a	Confirmed	
☐	☒	The exact sample size (n) for each experimental group/condition, given as a discrete number and unit of measurement
☐	☒	A statement on whether measurements were taken from distinct samples or whether the same sample was measured repeatedly
☐	☒	The statistical test(s) used AND whether they are one- or two-sided<br>*Only common tests should be described solely by name; describe more complex techniques in the Methods section.*
☐	☒	A description of all covariates tested
☐	☒	A description of any assumptions or corrections, such as tests of normality and adjustment for multiple comparisons
☐	☒	A full description of the statistical parameters including central tendency (e.g. means) or other basic estimates (e.g. regression coefficient) AND variation (e.g. standard deviation) or associated estimates of uncertainty (e.g. confidence intervals)
☐	☒	For null hypothesis testing, the test statistic (e.g. F, t, r) with confidence intervals, effect sizes, degrees of freedom and P value noted<br>*Give P values as exact values whenever suitable.*
☒	☐	For Bayesian analysis, information on the choice of priors and Markov chain Monte Carlo settings
☐	☒	For hierarchical and complex designs, identification of the appropriate level for tests and full reporting of outcomes
☐	☒	Estimates of effect sizes (e.g. Cohen's d, Pearson's r), indicating how they were calculated

Our web collection on statistics for biologists contains articles on many of the points above.

Software and code

Policy information about availability of computer code

Data collection	Electrophysiological data was collected with MC_Rack Multichannel Systems software v.4.6.2
Data analysis	Spyking circus software v1.06 was used for spike sorting. All data analysis and modelling was done in Python. The modelling package is available at https://gitlab.com/samuele_virgili/retina_modelling_2. The rest of the analysis code is available at https://github.com/samuelevirgili/Context-dependent-selectivity-to-natural-images-in-the-retina

For manuscripts utilizing custom algorithms or software that are central to the research but not yet described in published literature, software must be made available to editors and reviewers. We strongly encourage code deposition in a community repository (e.g. GitHub). See the Nature Portfolio guidelines for submitting code & software for further information.

Data

Policy information about availability of data

All manuscripts must include a data availability statement. This statement should provide the following information, where applicable:

- Accession codes, unique identifiers, or web links for publicly available datasets
- A description of any restrictions on data availability
- For clinical datasets or third party data, please ensure that the statement adheres to our policy

A minimum dataset (data from one example mouse experiment) generated in this study has been deposited in the Zenodo database at the link https://zenodo.org/record/6868362#.YtgeLoxBxH4 .

Due to the size of the full dataset, (hundreds of GBs), it has not been entirely uploaded in a public repository but authors are happy to share it upon request. Also, Source data for the figures panels 1e, 1f, 1g, 2b,2e,3e,4g are provided with this paper.

Human research participants

Policy information about studies involving human research participants and Sex and Gender in Research.

Reporting on sex and gender	*Use the terms sex (biological attribute) and gender (shaped by social and cultural circumstances) carefully in order to avoid confusing both terms. Indicate if findings apply to only one sex or gender; describe whether sex and gender were considered in study design whether sex and/or gender was determined based on self-reporting or assigned and methods used. Provide in the source data disaggregated sex and gender data where this information has been collected, and consent has been obtained for sharing of individual-level data; provide overall numbers in this Reporting Summary. Please state if this information has not been collected. Report sex- and gender-based analyses where performed, justify reasons for lack of sex- and gender-based analysis.*
Population characteristics	*Describe the covariate-relevant population characteristics of the human research participants (e.g. age, genotypic information, past and current diagnosis and treatment categories). If you filled out the behavioural & social sciences study design questions and have nothing to add here, write "See above."*
Recruitment	*Describe how participants were recruited. Outline any potential self-selection bias or other biases that may be present and how these are likely to impact results.*
Ethics oversight	*Identify the organization(s) that approved the study protocol.*

Note that full information on the approval of the study protocol must also be provided in the manuscript.

Field-specific reporting

Please select the one below that is the best fit for your research. If you are not sure, read the appropriate sections before making your selection.

☒ Life sciences ☐ Behavioural & social sciences ☐ Ecological, evolutionary & environmental sciences

For a reference copy of the document with all sections, see nature.com/documents/nr-reporting-summary-flat.pdf

Life sciences study design

All studies must disclose on these points even when the disclosure is negative.

Sample size	For the LSTAs experimental measure, all the neurons that after spike sorting showed less than 0.5% refractory period violations were used. For the modelling, across the 4 mice retinas and 2 axolotl retinas where the set of unperturbed inatural mages was presented, we selected the cells to be modeled under the following criteria: a) they did have a classical STA, b) for mouse recordings, whether their STA was stable across the experiment (assessed by repeating the checkerboard stimuli at the end) ; c) the spiking responses across the unperturbed, repeated images were stable. To assess stability, we used the criterion of Cadena et al. 13 and discarded the neurons that showed a ratio of explainable-to-total variance smaller than 0.10. We were able to model 90 cells in mice and 50 cells in axolotl.
Data exclusions	We extracted the activity of a total of 634 neurons from mice, and 50 neurons from axolotl. We kept cells with a low number of refractory period violations (<0.5%, with median <0.09% for all experiments, 2 ms refractory period) and whose template waveform could be well distinguished from the template waveforms of other cells. These constraints ensured a good quality of the reconstructed spike trains.
Replication	To guarantee replication, we describe in detail all the setup parameters, all the experimental protocols, the animal strains and age used and the methods to collect and analyze the data. We performed experiments on 6 mouse retinas and 2 axolotl retinas and we were always able to replicate the results.
Randomization	In our type of study we do not separate the data in groups. All the data from all the neurons across experiments is used in a single group.
Blinding	See the point above

Reporting for specific materials, systems and methods

We require information from authors about some types of materials, experimental systems and methods used in many studies. Here, indicate whether each material, system or method listed is relevant to your study. If you are not sure if a list item applies to your research, read the appropriate section before selecting a response.

Materials & experimental systems

n/a	Involved in the study
☒	☐ Antibodies
☒	☐ Eukaryotic cell lines
☒	☐ Palaeontology and archaeology
☐	☒ Animals and other organisms
☒	☐ Clinical data
☒	☐ Dual use research of concern

Methods

n/a	Involved in the study
☒	☐ ChIP-seq
☒	☐ Flow cytometry
☒	☐ MRI-based neuroimaging

Animals and other research organisms

Policy information about studies involving animals; ARRIVE guidelines recommended for reporting animal research, and Sex and Gender in Research

Laboratory animals	Data were recorded from isolated retinas from 6 C57BL6J mice of 8 to 11 weeks (median of 9, both male and female), and 2 adult female axolotl salamanders (Ambystoma mexicanum, pigmented wild-type). Extra 3 experiments were done for review purposes in 3 mice of 17 weeks.
Wild animals	No wild animals were used in the study.
Reporting on sex	The axolotls used were all females (not intended) but the mice used were both males and females and we did not find any change in the results depending on sex. Therefore, we did not differentiate for sex when reporting the results.
Field-collected samples	the study did not involve samples collected from the field.
Ethics oversight	The experiment was performed in accordance with institutional animal care standards of Sorbonne Université.

Note that full information on the approval of the study protocol must also be provided in the manuscript.
